# First record of an Icacinaceae Miers fossil flower from Le Quesnoy (Ypresian, France) amber

**DOI:** 10.1038/s41598-017-11536-y

**Published:** 2017-09-11

**Authors:** Cédric Del Rio, Thomas Haevermans, Dario De Franceschi

**Affiliations:** 1 Centre de Recherche sur la Paléobiodiversité et les Paléoenvironnements, CR2P- UMR 7207, CNRS, MNHN, UPMC, Muséum National d’Histoire Naturelle, Sorbonne-Universités, CP38, 57 rue Cuvier, 75231 Paris Cedex 05, France; 2 Institut de Systématique, Évolution, Biodiversité, ISYEB- UMR 7205, CNRS, MNHN, UPMC, EPHE, Muséum National d’Histoire Naturelle, Sorbonne-Universités, CP39, 57 rue Cuvier, 75231 Paris Cedex 05, France

## Abstract

Flowers embedded in amber are rare. Only about 70 flowers or inflorescences have been described among which only one lamiid is known. Nevertheless, these fossils are important to our understanding of evolutionary process and past diversity due to the exceptional preservation of fragile structures not normally preserved. In this work, a new flower named *Icacinanthium tainiaphorum* sp. nov. from Le Quesnoy (Houdancourt, Oise, France) is described. Our phylogenetic analysis with extant species suggests that the affinity of this flower lies with the family Icacinaceae, close to *Natsiatum* or *Hosiea*. The fossil shows a combination of features unknown in extant Icacinaceae and we thus propose the description of a new fossil genus. It reveals a previously unknown diversity in the family and demonstrates the complementarity of different types of fossil preservation for a better understanding of past floral diversity.

## Introduction

Among the diverse organisms or parts of organisms embedded in amber, angiosperm flowers are exceedingly rare. Only ca. 70 flowers or inflorescences have been described to date, mostly from Cretaceous^1^ Burmese amber^2–4^, Paleogene Baltic amber^5, 6^ Miocene^7^ Dominican amber^8–18^ and Late Oligocene-Early Miocene Mexican amber^19–21^.

In addition, we are noticing phylogenetic disparities. The rosid clade accounts for 39% of eudicot diversity but 44% of flowers embedded in amber. In contrast, the asterid clade, which represents half of the eudicot diversity^22^, is represented in the fossil record by only 12 flowers (17%) in amber. Among these flowers, ca. 50% belong to Ericales and Cornales. Whilst five flowers belong to campanulids, only one represents the lamiid clade (in the order Gentianales), which corresponds to 26% of the extant eudicot diversity. Nevertheless, fossil flowers embedded in amber are generally important to understand evolutionary processes and past diversity because they are often exceptionally well preserved and provide insights into the morphology of fragile structures that are not normally preserved^23^.

The locality at Le Quesnoy (Houdancourt, Oise, France), considered to be basal Eocene (Ypresian) was first studied in 1999^24^. This first survey highlighted the abundance of insects and the presence of at least one flower of caesalpinioid legume in amber. The pollen included in the amber was studied using a new technique of extraction^25, 26^, but the diversity of the floral remains had to be studied extensively.

Here we study one flower from Le Quesnoy and assign it to the lamiid clade. This flower named *Icacinanthium tainiaphorum* sp. nov. is the first known fossil occurrence of a flower from order Icacinales. The affinity of this flower embedded in the resin of a tree might suggest that this species could have been a climber. This type of ecology is frequent in megathermal flora, which has developed in Europe during the Eocene global warming. This Icacinaceae flower in amber attests to the presence of an undocumented past diversity.

## Results

### Systematics

Order Icacinales Tiegh.

Family Icacinaceae Miers.

Genus *Icacinanthium* Del Rio & De Franceschi, gen. nov.

Type species *Icacinanthium tainiaphorum* Del Rio & De Franceschi, sp. nov.

Generic diagnosis: Flower small, actinomorphic, hypogynous and pentamerous. Calyx cupular, Petals lanceolate curved backwards, with a straight apex, fused at base in a short cup, tomentose on adaxial surface with long, simple flattened hairs with granular ornamentation, glabrous on abaxial surface. Stamens alternate to petals, free. Pollen small, triaperturate and echinate.

Etymology: “*Icacinanthium*” indicates the familial affinity to Icacinaceae (Icaci-) and that it is a flower (L = Anthos, flower).

**Figure 1:**
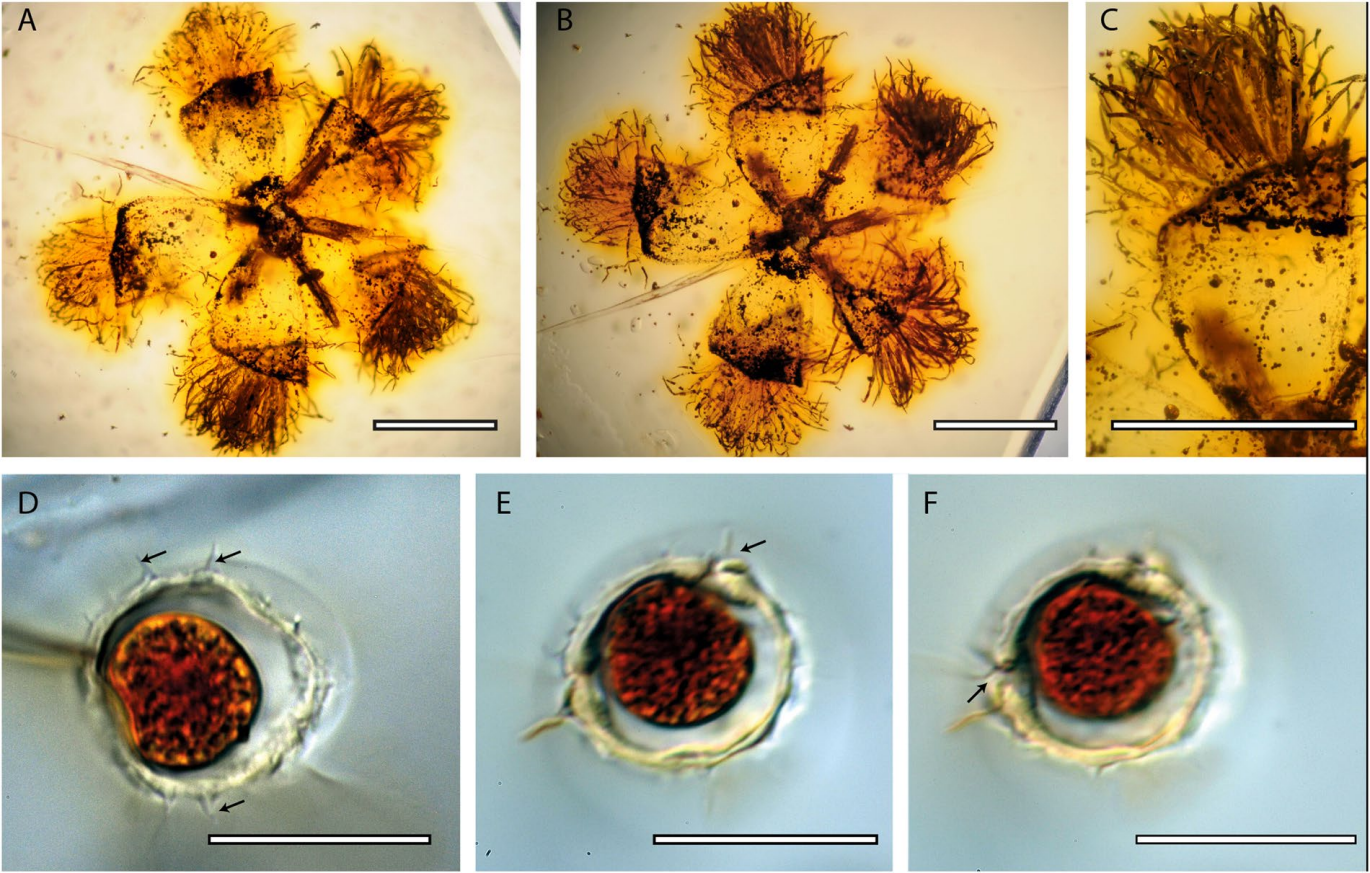
*Icacinanthium tainiaphorum* holotype, (**A**) flower in apical view with petals curved backwards, (**B**) same flower in basal view, (**C**) detail of petals in basal view (**D**) pollen grain showing echinate ornamentation (arrows) (**E** and **F**) other pollen grain showing pores (arrows). Scale: (**A**–**C**) = 1mm, (**D**–**F**) = 20 µm.

**Figure 2:**
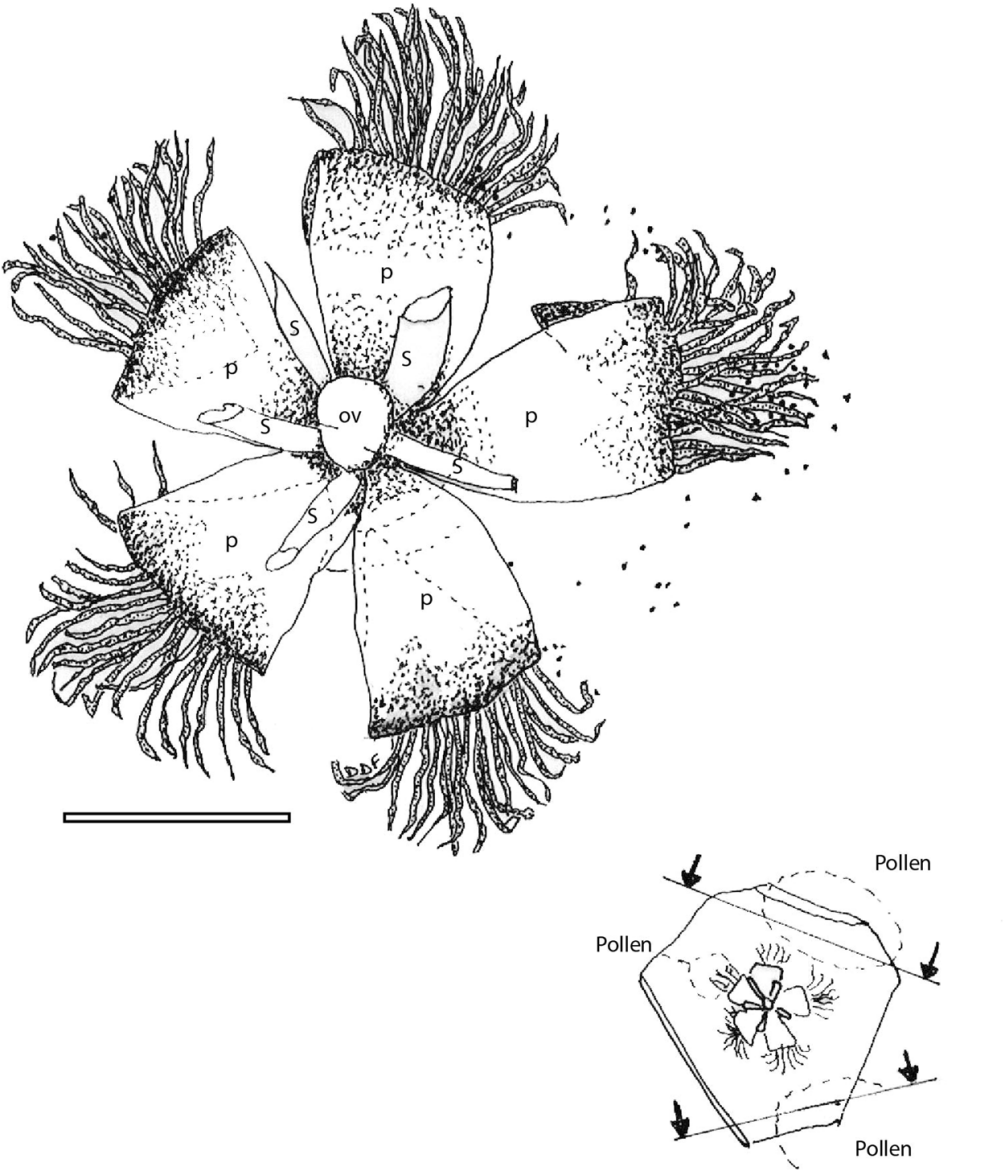
Drawing of *Icacinanthium tainiaphorum* gen. et sp. nov. in apical view; (ov) ovary; (s) stamens; (p) petals. Arrows and lines: position of section for pollen preparation. Scale bar = 1mm.

*Icacinanthium tainiaphorum* Del Rio & De Franceschi, sp. nov. (Figs 1 and 2)

Holotype: Deposited in the collection of Palaeobotany of MNHN (MNHN.F.44051.)

Type Locality: Le Quesnoy, Oise, France

Stratigraphic age: Ypresian

Etymology: “*tainiaphorum*” refers to the flat “ribbon-like” (L = tainia, ribbon) hairs borne (L = phor, bearing) on the petals.

Specific diagnosis: As for the genus

Description: A single flower embedded in amber, almost completely preserved. Flower small, about 2.5 mm in diameter, at least unisexual male or hermaphrodite, actinomorphic pentamerous and hypogynous (Fig. 1A,B).

The calyx is cupulate, filmy and very short. It is composed of 5 minute sepals, which are very difficult to distinguish. Scattered single-celled hairs are visible at the margin. Aestivation of the corolla is probably valvate according to the shape and position of the petals, which are fused at the base in a short cup. The petals are lanceolate, curved backwards in apical view (Figs 1A and 2) and with a straight apex (Fig. 1C), about 1.40 mm long and 0.7 mm broad. They are tomentose on the adaxial surface with long, simple flattened hairs with granular ornamentation, about 0.7 mm long and 0.025–0.036 mm broad; The stamens alternate with the petals and are erect but anthers were not preserved; Filaments are glabrous, about 0.15 mm in diameter, free from the petals and attached below the base of gynoecium. The globular gynoecium is free, possibly unilocular considering its shape, and glabrous. The ovary seems to be poorly developed or very small. The stigma is unknown, not preserved or not developed.

The pollen is very small (about 20–25 µm in diameter), clearly echinate (Fig. 1D) and triporate (Fig. 1E,F). No colpus could be observed, but we cannot completely exclude the presence of faintly marked small colpi.

### Phylogenetic analysis

**Figure 3:**
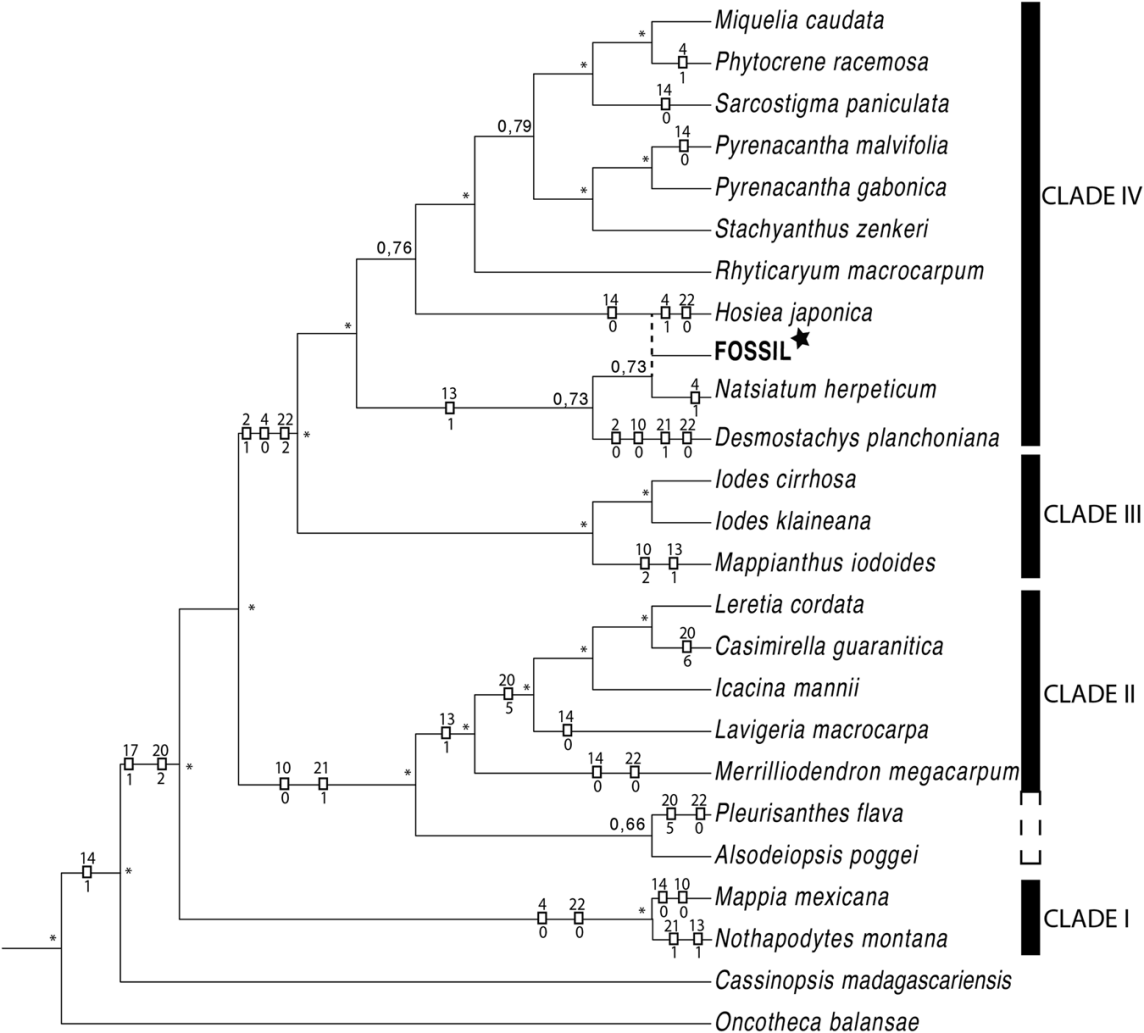
Phylogenetic relationships among asterid species focused on the family Icacinaceae based on the combined 73-plastidial genes and 22 morphological characters. The 50% majority-rule consensus tree was constructed by Bayesian inference in MrBayes. Star indicates the placement of the fossil flower close to *Natsiatum*. Dotted lines indicate another possible position for the fossil, close to *Hosiea*. Mesquite orientation of pertinent characters is shown. (*) represent strong node (>95% PP).

The Bayesian analysis 50% majority-rule consensus tree is given in Fig. 3 (and Supplementary 1). We included 51 species whose 23 species represent 21 genera of Icacinaceae and 27 species represent all other groups of the asterid. We used a combined morphological and molecular data with 73 protein-coding genes for a total of 59132 bp and 22 morphological characters (see the material and method for more precisions). As expected, we found a topology similar to the previous phylogenetic study using the same DNA data for the species included here^27^. This fossil, when included in the analyses, is placed close to *Natsiatum*. However the node with *Icacinanthium* and *Natsiatum* have very low support (PPB < 0.95). In this sense, the position of *Icacinanthium* is considered as unresolved at the base of the group IV sensu Stull *et al*.^27^.

One hundred and ninety five (195) steps are necessary for the reconstruction of the history based on morphological characters by Mesquite’s parsimony state reconstruction (0.34 CI and 0.47 RI). The position of *Icacinanthium* close to *Natsiatum* or *Hosiea* is supported by the same number of steps. This explains why only the nodes of the clades containing *Natsiatum* and *Hosiea* are over 0.95 PP. Of the 22 morphological characters, only the stamen position [16] and the symmetry of the flower [1] are non informative (Fig. 3; Supplementary 2). For the fossil flower, the main informative characters are the calyx shape [4], the petal and stamen fusion [10; 17] and all pollen characters [20; 21; 22]. Positioning uncertainties correspond to both characters for presence or absence of hair adaxially and abaxially on petals [13; 14].

## Discussion

Only the presence of hairs on the outside of the petals seems to be synapomorphic for the Icacinaceae group (Fig. 3) according to our phylogenetic reconstruction. While *Icacinanthium* did not have hairs outside the petals, this state is also absent in four genera and some species of other genera within Icacinaceae, indicating a convergent loss of these hairs.

According to our ancestral state reconstruction, the Icacinaceae clade (excl. *Cassinopsis*) possesses small bisexual, pentamerous flowers with petal apex adaxially curved, stamen free from petals (this last state shared by all modern genera of Icacinaceae s.s, sensu Stull *et al*.^27^) and echinate pollen. Only the shape of the petal apex does not match with this fossil flower; however, this feature also occurs in five extant genera of Icacinaceae and several species which also possess comparable straight apices.

The clade including both III and IV possesses unisexual flowers (except for *Desmostachys*), cupular calyx, petals fused at base and small and mainly porate pollen grains (synapomorphic). Moreover, petal apex orientation of Clade IV is unresolved with a preponderance of genera with straight apices, which would be a synapomorphic character for this clade, emphasizing affinities of *I*. *tainiaphorum* to this clade. We therefore hypothesize that this flower is a unisexual male flower, which would explain the poorly developed ovary and make a better fit with the phylogenetic position of the fossil (rather than a poor preservation of this organ during fossilisation).

**Figure 4:**
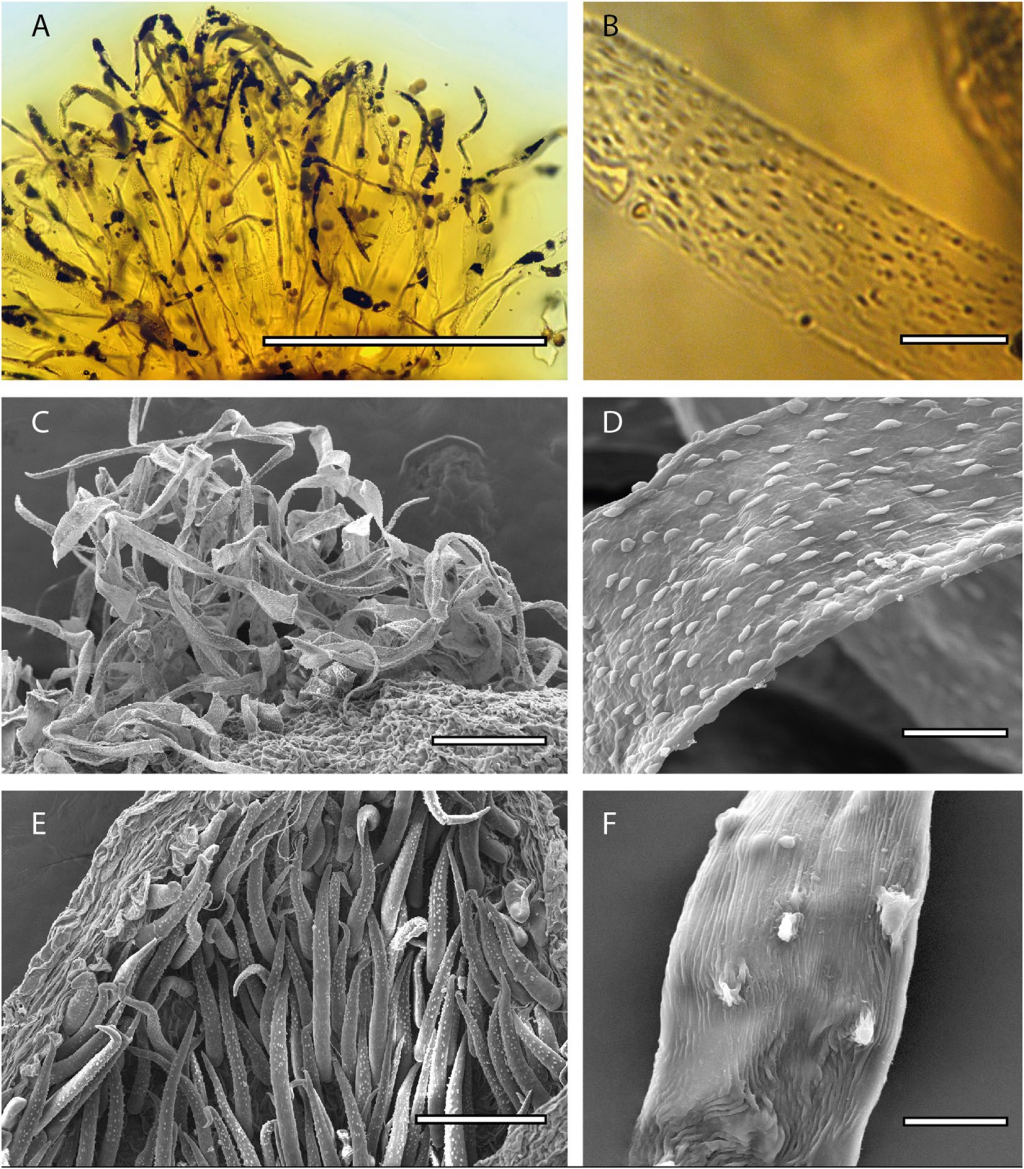
Morphological form of hairs on petals. (**A**) “simple” adaxial hairs from *Icacinanthium tainiaphorum*, note the presence of pollen grains between hairs (**B**) same, detail of ornamentation on hair surface (**C**) “simple” adaxial hairs from *Icacina mannii* Oliv.; (**D**) same, detail of ornamentation (**E**) tubular and acute abaxial petal hairs from *Icacina mannii*. (**F**) detail of ornamentation for *Metteniusa tessmaniana* Scale: (**A**) = 500 µm (**C** and **E**) = 100 µm; (**B**,**D** and **F**) = 20 µm.

Adaxial hairs of *Emmotum nitens* ^28^ and *Poraqueiba sericea* have an irregularly moniliform shape, and thus clearly differ from the hairs seen in other species. In fact, all adaxial hairs of Icacinaceae s.s. considered here and *Metteniusa* petals are flattened and very simple, as what is found in *Icacinanthium* (Fig. 4). Icacinaceae hairs however are granular, more or less longitudinally extended while in *Metteniusa* we observe nodules surrounded by longitudinal furrows (Fig. 4F), very different from the Icacinaceae ornamentation. These observations corroborate the position of *I*. *tainiaphorum* within the family Icacinaceae. In this family, the external hairs are generally quite similar, with a tubular form, acute at the apex and with granular ornamentation (Fig. 4E). The hairs in the fossil appear to be homologous and the presence of these hairs is interpreted to be synapomorphic for Icacinaceae.

In any case this fossil shows a combination of characters unknown in extant Icacinaceae. Indeed, flowers of *Natsiatum herpeticum*, which have adaxial petal hairs, also have them abaxially. In contrast, *Hosiea japonica* flowers are glabrous, and there are also flowers with abaxially hairy petals, but not adaxially in four genera of clade IV. To our knowledge, only the flower of *Merrilliodendron megacarpum*, from clade II, has adaxial petal hairs only such as what we found in our fossil flower. In clade IV this character state is unknown and new, hence our proposal to treat it as an extinct genus.

The fossil record of Icacinaceae, predominantly from the Eocene of North America and Europe, is extensive. However no fossil flowers were until now attributed to this family. In fact, most of these fossils are endocarps, in particular from the modern genera *Iodes* ^29–31^, *Phytocrene* ^32, 33^ and *Pyrenacantha* ^30, 33^. Endocarp fossils of *Natsiatum* were described from the Middle Eocene of Tennessee (*Natsiatum wilcoxiana* (Berry) Stull, Moore & Manchester^34^). In Europe, fossils of *Palaeohosiea*, supposed to be close to the extant genus *Hosiea*, were described^35^. However, no character supports affinity with *Hosiea* and no feature separates the fossils from the extant genus *Iodes* ^36^. The presence of Icacinaceae from Le Quesnoy was only mentioned briefly and was based on what appears to be a lignitic endocarp^24^, but was attributed to the genus *Iodes* (personal observation). The link between these two types of fossils is questionable, thus indicating an unsuspected diversity for Eocene Icacinaceae.

Growth habits of extant Icacinaceae include lianas, shrubs or trees, distributed in tropical forests around the word. *Icacinanthium* is close to *Natsiatum* or *Hosiea*, both of which being Asian genera of climbing shrubs^37^. *Icacinanthium* could testify for an Asian affinity of the Le Quesnoy Ypresian flora, as highlighted by studies on Menispermaceae^38^ and other comparable European sites^31, 32, 39^. We thus hypothesize that *Icacinanthium* could be a climbing shrub, this type of ecology being frequent for megathermal flora that would have occurred in Europe during the early Eocene global warming phase^40, 41^.

## Material and Method

### Locality and fossil material

The fossil resin remains were collected from 1997 to 2000 from the Le Quesnoy (Houdancourt, Oise, France) lignitic clay sediments which belong to the ≪ argiles à lignites du Soissonais ≫ Formation. These sediments are dated to the Ypresian (±56 Ma) according to mammal biochronology (MP7) and palynological studies^24, 42^. This corresponds to the Sparnacian facies of the lower Ypresian (lower Eocene). Several fossil resin samples contain diverse organisms, principally arthropods (mainly insects), a few plant remains, mainly represented by pollen grains^25, 42^ but also a few flowers still to be studied. The fossils are kept in the collections of the Paris Muséum national d’histoire naturelle (MNHN). The specimen studied here is a flower embedded in this fossil resin here called amber, but produced by Detarioidae trees (Leguminosae)^43, 44^.

### Phylogenetic reconstructions

Preliminary exploratory herbarium studies (P) focusing on small pentamerous hypogynous flower with petals fused at the base and with stamens free from the petals lead to comparison of the flower in amber to those of the family Icacinaceae. We employed the most complete molecular data for the asterid clade focusing on the Icacinaceae^27^. Theses data were composed of 73 protein-coding genes for a total of 59132 bp. We retained 51 species among the 112 accessions available^27^ of which 23 species represent 21 genera of Icacinaceae. We included at least 2 species from other asterid orders, except for Metteniusales where we used 9 species as this order includes species which were formerly included in genera of Icacinaceae, which are morphologically close to Icacinaceae sensu stricto. All molecular characters are coded as missing for the flower in amber. In addition, we included 22 morphological floral characters corresponding to features potentially observable in the fossil flower (Supplementary 2). The final matrix contains 59135 characters.

Bayesian analyses were performed using MrBayes^45^ with a format type mixed with two partition: DNA type (with model by default, GTR + I + G) and Standard type (equal state frequencies with all topologies equally likely a priori with unconstrained branch lengths) for morphological data. Two independent but parallel analyses were performed using flat priors, starting from random trees and consisting of four chains each. The analysis was run for 5 million generations, sampling every 1000 generations and with 20% burn-in. Analysis of output parameter using Tracer v.1.6^46^ confirms the convergence of chains. A 50% majority-rule consensus tree was computed with posterior probability (PP) estimates for all nodes. We generally consider as non-supported nodes those with less than 0.95 PP.

### Morphological studies

A morphology matrix was made using Xper 3^47^ containing 22 flower and pollen characters coded for 51 taxa including the Le Quesnoy flower (Supplementary 2)

The matrix was constructed from direct morphological observation of herbarium specimens (P, see Supplementary 3) and from bibliographic data for flowers^28, 37, 48–57^ and pollen^58–63^. We observed the flower embedded in amber using a light microscope (Nikon Eclipse80i). Numerous pollen grains were found around the flower and between hairs on the adaxial surface of the petals (Fig. 1D,F); the vicinity and abundance of grains of the same type indicate that they probably belong to the flower. The pollen was also extracted from the amber in order to obtain a better understanding of its type (Fig. 2) following a previously published protocol^25^ and observed using the same microscope. Before extraction we only observe the imprint left by the pollen in amber. After extraction, we show an exceptional preservation of grains with well-preserved exine wall and remains of cellular content. A darker zone could correspond to the rest of the nucleus (clearly visible on Fig. 1D). Due to the presence of echinae (spines), pollen grains could not be fully separated from resin and were observed still embedded inside a small resin block. All these features were mapped into the phylogeny using Mesquite software^64^ with a parsimony ancestral state reconstruction.

We conducted a qualitative study of adaxial petal hairs of *Metteniusa*, *Poraqueiba*, *Nothapodytes*, *Mappia*, *Icacina*, *Leretia*, *Mappianthus*, *Desmostachys* and *Natsiatum* using SEM (Scanning Electron Microscopy) with a Jeol JCM6000 after the specimens were coated with gold-palladium.

## Electronic supplementary material

Supplementary Information
