# Supplementary material for: First record of an Icacinaceae Miers fossil flower from Le Quesnoy (Ypresian, France) amber
Source: Sci Rep. 2017 Sep 11;7:11099. doi: 10.1038/s41598-017-11536-y (PMC5593952; doi:10.1038/s41598-017-11536-y)
Supplement: Supplementary file 1 — Supplementary Information [file 41598_2017_11536_MOESM1_ESM.doc]

First record of an Icacinaceae Miers fossil flower from Le Quesnoy (Ypresian, France) amber

Cédric Del Rio^1, 2, *^, Thomas Haevermans^2^, Dario De Franceschi^1^

^1^ UMR7207, CR2P, MNHN-CNRS-UPMC, Sorbonne-Universités, CP38, 8 rue Buffon, 75231 Paris Cedex 05, France

^2^ UMR7205, ISYEB, CNRS-MNHN-UPMC-EPHE, Sorbonne-Universités, CP50, 45 rue Buffon, 75231 Paris Cedex 05, France

^*^Correspondence to [cedric.del-rio@edu.mnhn.fr](mailto:cedric.del-rio@edu.mnhn.fr)

Running Head: Icacinaceae flower in Le Quesnoy amber

**Supplementary 1.** Phylogenetic relationships among asterid species focused on the family Icacinaceae based on the combined 73-plastidial genes and 22 morphological characters. The 50% majority-rule consensus tree was constructed by Bayesian inference in MrBayes.

**Supplementary 2.** Flower and pollen morphological matrix.

#NEXUS

[!Export generated by Xper3 on 2017-04-27 16:40:12]

Begin Data;

Dimensions NTax=51 NChar=22;

Format Missing=? Gap=N Symbols="0 1 2 3 4 5 6 7 8 9 a b c d e f g h i j k l m n o p q r s t u v w x y z ";

Options GapMode=Missing;

CharStateLabels

1 Flower_symmetry/ Bilateral Radial,

2 Flower_sex/ Bisexual Unisexual,

3 Flower_size/ Small Tall,

4 Calyx_form/ Cupular Divided,

5 Sepal_number_of_pieces/ 0 4 5 6 8,

6 Sepals_hair_on_margin/ Yes No,

7 Petal_number/ 4 5 6 8,

8 Petal_aestivation/ Imbricate Twisted Valvate,

9 Petal_shape/ Circular Lanceolate Oblong_to_linear Spatulate Triangular,

10 Petals_welding/ Free Gamopetal_at_base Gamopetal_in_tube,

11 Petal_apex_orientation/ Curved_adaxially Straight,

12 Petals_orientation_in_flower/ Curved_abaxially Straight,

13 Petals_hair_inside/ Absent Present,

14 Petal_hair_outside/ Absent Present,

15 Stamen_number_of_pieces/ 2 4 5 6 8 10,

16 Stamen_position/ Alternate_with_petals Opposite_with_petals,

17 Stamen_fusion/ Adnate_to_petals Free,

18 Ovary_hair/ Absent Present,

19 Ovary_locule/ Plurilocular Unilocular,

20 Pollen_ornamentation/ Baculate Clavate Echinate Gemmate Granulate Reticulate Scabrate Verrucate,

21 Pollen_size/ Small Tall,

22 Pollen_aperture/ Colpate Colporate Porate

;

Matrix

Alsodeiopsis_poggei 1001201210010120111211

Apodytes_dimidiata 1000211220100020101501

Aucuba_japonica 11001102201100101110(01)1

Borago_officinalis 1011201241110020000311

Calatola_mollis 11001?0220???110011701

Casimirella_guaranitica 1001(23)0(12)2(12)01111(23)?111701

Cassinopsis_madagascariensis 1001201221110120011501

Coffea_arabica 1010(123)111221100200?14(01)1

Convolvulus_arvensis 1011211022110020000410

Cornus_florida 10000002210101101?0??1

Daucus_carota (01)000(02)11?20010020100401

Desmostachys_planchoniana 10002012201?1120111210

Ehretia_acuminata 1001201022110020000?01

Emmotum_nitens 1000201220111120010601

Garrya_flavescens 110?0102(12)11?01?0110511

Gentiana_acaulis 1011211122100020001501

Hosiea_japonica 11012?12111000201012(01)0

Hydrolea_corymbosa 10112010211100201105?1

Icacina_mannii 1001201220011120111511

Ilex_cornuta 1101100021110010100101

Iodes_cirrhosa 110(01)2012(12)1010120111202

Iodes_klaineana 1100201210110120111202

Jasminum_nudiflorum 1011(23)1(12)022110000000510

Lavigeria_macrocarpa 1001201210010020111511

Le_Quesnoy 1?002012111010201??202

Leretia_cordata 10012012(12)0011120111511

Mappia_mexicana 1000201210010020101200

Mappianthus_iodoides 11002012220111201112(01)2

Merrilliodendron_megacarpum 10012112200?1020101210

Metteniusa_tessmanniana 1011201021101020011??1

Miquelia_caudata 1100(12)?(01)2?10???(12)??11202

Natsiatum_herpeticum 1101201211101120101202

Nothapodytres_montana 1000201221001120111210

Oecopetalum_mexicanum 10(01)0201?(12)???1020?11601

Oncotheca_balansa 1001211001110020000501

Ottoschulzia_rhodoxylon 1000201221100120101601

Phytocrene_racemosa 11012002(12)1110110111202

Pittosporopsis_kerrii 1001201230110120001501

Platea_latifolia 1101201221110020001501

Pleurisanthes_flava 10012012(12)0010120111510

Poraqueiba_sericea 100121122001112?101601

Pyrenacantha_gabonica 110???02211101101??202

Pyrenacantha_malvifolia 110???(01)2411100(12)0111202

Rhaphiostylis_ferruginea 11(01)0201220100020111501

Rhododendron_simsii 1011201022110150110411

Rhyticaryum_macrocarpum 11002?12(12)1010120111202

Sarcostigma_paniculata 11002012210100201112(01)2

Solanum_lycopersicum 1011211221100020000?01

Stachyanthus_zenkeri 110030222?01013???1202

Thymus_vulgaris 0011201?(02)2110110000510

Vahlia_capensis 10112012(01)11110201015?1;

End;

Begin Assumptions;

Options DefType = Ord;

End;

**Supplementary 3.** Modern flowers examined for this study.

Herbarium samples examined for comparison are listed below, including the following information: voucher number, locality, date of collection and bar code of herbarium (P). The specimens used for hair comparison are in bold.

**Boraginales.** *Borago officinalis* L., Bosserdet SN, France, 1937, P04026682; *Ehretia acuminata* R. Br., Desvaux SN, Nouvelle-Zelande, *, P03860204. **Cornales.** *Cornus florida* L., Vaillant SN, *, 1945, P04556278. **Ericales.** *Rhododendron simsii* Planch., Poilane 29733, Indochine, 1939, P04505977. **Garryales**. *Aucuba japonica* Thunb., Savatier 531, Japan, *, P00545427; *Garrya flavescens* S. Watson, Purpus 841, California, 1894, P04472060.

**Gentianales.** *Coffea arabica* L., Meyer 781, Ethiopia, 1961, P03826715 ; *Gentiana acaulis* L., *, France, 1962, P05039952. **Icacinales.** *Alsodeiopsis poggei* Engl., Letouzey 11516, Cameroun, 1972, P04495898 ; Cassinopsis madagascariensis Bail., Phillipson et al. 5643, Madagascar, 2003, P04472155 ; *Desmostachys planchoniana* Miers, Randrianarivelo et al. 62, Madagascar, 2004, P00692577 ; *Desmostachys tenuifolius* Oliv., Endengle 141, Cameroun, 1960, P04495143 ; *Icacina manii* Oliv., *, Afrique occidentale, *, P04495581 ; *Iodes cirrhosa* Turcz., *, Sud Vietnam, 1960, P06672326 ; *Iodes cirrhosa* Turcz., Poilane 9956, « Indochine », 1924, P06672329 ; *Iodes klaineana* Pierre, Sita 2571, Congo, 1968, P04472290 ; *Lavigeria macrocarpa* (Oliv.) Pierre, Le Testu 4487, Yalinga, 1923, P04494816; *Lavigeria macrocarpa* (Oliv.) Pierre, Tisserant 2354, Bouhko, 1976, P04494814; *Leretia cordata* Vell., B. 4445, Guyane, 1972, P06672311; *Mappia mexicana* B.L. Rob. & Greenm, *, Tampico, 1897, P04513549; *Mappia racemosa* Jacq., Manriquez 3472, Mexico, 1990, P04513546 ; *Mappianthus iodoides* Hand.-Mazz, *, Tonkin, 1944, P04513536 ; *Mappianthus iodoides* Hand.-Mazz, Petelot 8750, Tonkin, 1943, P04513537 ; *Natsiatum herpeticum* Buch.-Ham. ex Arn., Bena 1142, Guadeloupe, *, P04519509 ; *Natsiatum herpeticum* Buch.-Ham. ex Arn., Griffith 170, Inde, 1850, P04513508 ; *Nothapodytes montana* Blume, Beusekom & Phengkhlai 48, Thailand, 1968, P04513563 ; *Nothapodytes pittosporoides* (Oliv.) Sleumer, *,*,*, P04513555 ; *Oncotheca balansae* Bail., Suprin 1601, Nouvelle-Calédonie,1981, P00181264 ; *Pleurisanthes flava* Sandwith, Werff & Vasquez 13898, Peru, 1995, P05279709 ; *Sarcostigma paniculata* Pierre, Pierre 1644, Vietnam, 1877, P00834244 ; *Stachyanthus zenkeri* Engl., Zenker 4914, Cameroun, 1913, P05030980. **Lamiales.** *Thymus vulgaris* L., Didier 1016, France, 1949, P04436090; **Metteniusales.** *Emmotum nitens* (Benth,) Miers, Irwin et al. 27399, Brasil, 1970, P04513263; *Metteniusa tessmanniana* (Sleumer) Sleumer, Asplund 10211, Equator, 1940, P06590944; *Pittosporopsis kerrii* Craib, Poilane 20731, Laos, 1969, P04464735; *Platea latifolia* Blume, Liang 65285, Hainan, 1934, P06669774. *Poraqueiba sericea* Tul., Sastre 2476, Colombie, 1973, P05279040; **Solanales.** *Hydrolea corymbosa* Elliott, Howard 12951, Florida, 1952, P04054102; *Solanum lycopersicum* L., Fosberg et al. 28204, Peru, 1947, P03961429.
